# Percutaneous Closure of Patent Foramen Ovale is Associated With Improvement of Migraine

**DOI:** 10.31083/RCM44499

**Published:** 2026-03-06

**Authors:** Xiao-Ming Ge, Kang-Ning Han, Fei Gao, Zhi-Jian Wang, Yu-Jie Zhou

**Affiliations:** ^1^Department of Cardiology, Beijing Anzhen Hospital, Capital Medical University, 100029 Beijing, China; ^2^Department of Cardiology, The First Hospital of Fangshan District, 102400 Beijing, China

**Keywords:** patent foramen ovale, migraine disorders, heart septal defects

## Abstract

**Background:**

Patent foramen ovale (PFO) is the most common congenital heart defect and has been linked to migraines; however, the relationship between PFO and migraine remains controversial. This study aimed to evaluate whether percutaneous PFO closure alleviates migraines and explore the association between PFO and migraine.

**Methods:**

Data from 5581 inpatients with PFO were collected between 2015 and 2020. A total of 71 stroke-free adults with PFO (45 with closure and 26 without) were included. Self-reported migraine history, frequency, and severity (0–10) were assessed. Outcomes were compared between patients with and without PFO closure, and logistic regression was used to examine the relationship between PFO closure and migraine improvement.

**Results:**

PFO closure significantly reduced migraine frequency and severity, with greater improvements observed after 2 years (*p* *<* 0.001). Logistic regression showed that PFO closure was associated with a higher likelihood of migraine improvement than non-closure (odds ratio (OR): 5.57, 95% confidence interval (CI): 1.76–17.68; *p* = 0.004). This association persisted after adjusting for multiple risk factors (*p* = 0.005).

**Conclusion:**

Percutaneous PFO closure significantly improved migraines by reducing both frequency and severity, supporting a potential association between PFO and migraine.

## 1. Introduction

Patent foramen ovale (PFO), a remnant of fetal circulation, is present in approximately 25% of the general population [1, 2, 3] and represents the most common congenital cardiac defect. It allows blood and other substances from the right atrium to bypass the pulmonary circulation and enter the left atrium directly [4]. Although many individuals with PFO are asymptomatic, studies have shown associations with cryptogenic stroke, decompression illness, platypnea-orthodeoxia, and migraine [5, 6, 7]. The prevalence of PFO is significantly higher among migraineurs, and patients with PFO are more likely to experience migraines [8, 9].

Subclinical thrombi and vasoactive substances, such as serotonin, may pass directly from the right heart into systemic circulation through a PFO. Normally metabolized in the lungs, serotonin can directly irritate the trigeminal nerve and trigger migraine attacks. It may also promote platelet activation and aggregation, leading to further serotonin release [10]. Another study reported that subclinical microemboli traversing the PFO into cerebral circulation can occasionally cause bioelectrical disturbances, potentially contributing to migraine pathogenesis [7, 11]. PFO-related hypoxia and elevated plasminogen activator inhibitor-1 expression may suppress fibrinolysis and increase hypercoagulability, facilitating micro-emboli formation [12, 13].

PFO closure, primarily performed for stroke prevention, may also reduce migraine frequency and severity [14, 15]. Higher PFO prevalence has been reported in patients with both cryptogenic stroke and migraine [16, 17]. The proportion of PFO in patients with migraine and a history of stroke is significantly higher than in patients without stroke [18]. Patients with PFO who present with decompression illness or paradoxical embolism are also more likely to have migraines [19, 20]. Several studies have shown migraine improvement following PFO closure [21, 22, 23, 24, 25]. However, a cross-sectional study reported no significant difference in PFO prevalence between patients with self-reported migraine and those without [26]. Randomized trials of PFO closure have been conducted—two with sham procedures [27, 28] and one without [29]. All demonstrated numerical reductions in migraine burden by PFO closure, but improvements did not reach statistical significance for primary endpoints, only for certain secondary endpoints. Re-analysis of aggregated patient data from two trials using the Amplatzer PFO occluder, or redefining primary endpoints, demonstrated highly significant migraine improvement [30].

Given that migraines affect over 10% of the general population and pose a substantial public health burden, clarifying the association between PFO and migraine is clinically important [31]. Therefore, we aimed to compare migraine symptoms in patients with PFO who underwent percutaneous closure versus those who did not.

## 2. Methods

### 2.1 Patient Selection

This single-center, observational, retrospective study included inpatients with both PFO and migraine admitted to Beijing Anzhen Hospital between 2015 and 2020. Inclusion criteria were as follows: (a) diagnosis of migraine according to the International Headache Society [32], and (b) PFO confirmed by transcranial Doppler or transthoracic echocardiography. Exclusion criteria were as follows: (a) age <18 years; (b) missing clinical data; and (c) history of stroke, coronary artery disease, cancer, atrial fibrillation, myocardial infarction, or heart failure. Migraine information was obtained from patients or family members via telephone interviews. Patients reported migraine frequency within the past 3 months and rated severity on a 0–10 scale before and after hospitalization. Some patients without PFO closure received medications for migraine attacks. Migraine improvement was defined as a decrease in frequency and/or severity score.

### 2.2 Data Collection 

Demographic, clinical, and laboratory data were extracted from medical records. Demographic variables included age and sex. Vital signs included systolic blood pressure (SBP), diastolic blood pressure (DBP), and heart rate. Medical history included diabetes, hypercholesterolemia, and hypertension. Laboratory parameters included red and white blood cell counts, platelets, hemoglobin, fasting plasma glucose (FPG), low-density lipoprotein cholesterol (LDL-C), high-density lipoprotein cholesterol (HDL-C), total cholesterol (TC), triglycerides (TG), creatinine, alanine transaminase, and aspartate transaminase. Body mass index (BMI) was calculated as weight (kg) divided by height squared (m^2^).

Hypertension was defined as SBP ≥140 mm Hg, DBP ≥90 mm Hg, or use of antihypertensive drugs. Diabetes was defined as FPG ≥7.0 mmol/L or use of antidiabetic drugs. Dyslipidemia was defined as a fasting serum TC >5.17 mmol/L, LDL-C >3.36 mmol/L, TG >1.69 mmol/L, HDL-C <1.03 mmol/L, or chronic use of lipid-lowering drugs.

### 2.3 Statistical Analysis

Continuous variables with normal distribution were expressed as mean ± standard deviation and compared using independent or paired sample *t*-tests. Non-normally distributed variables were expressed as medians (25th–75th percentiles) and analyzed using the Mann–Whitney test. Categorical variables were presented as percentages and compared using Pearson’s chi-square (χ^2^) or Fisher’s exact test.

Logistic regression analyses were conducted to evaluate the association between PFO closure and migraine improvement. The following three multivariate models were applied: (a) Model 1, unadjusted; (b) Model 2, adjusted for age and sex; and (c) Model 3, adjusted for age, sex, and diabetes.

Receiver operating characteristic (ROC) curves were used to evaluate the sensitivity and specificity of PFO closure for predicting migraine improvement. All analyses were performed using SPSS version 26 (SPSS Inc., Chicago, IL, USA). Statistical significance was set at *p* < 0.05.

## 3. Results

**Fig. 1.**
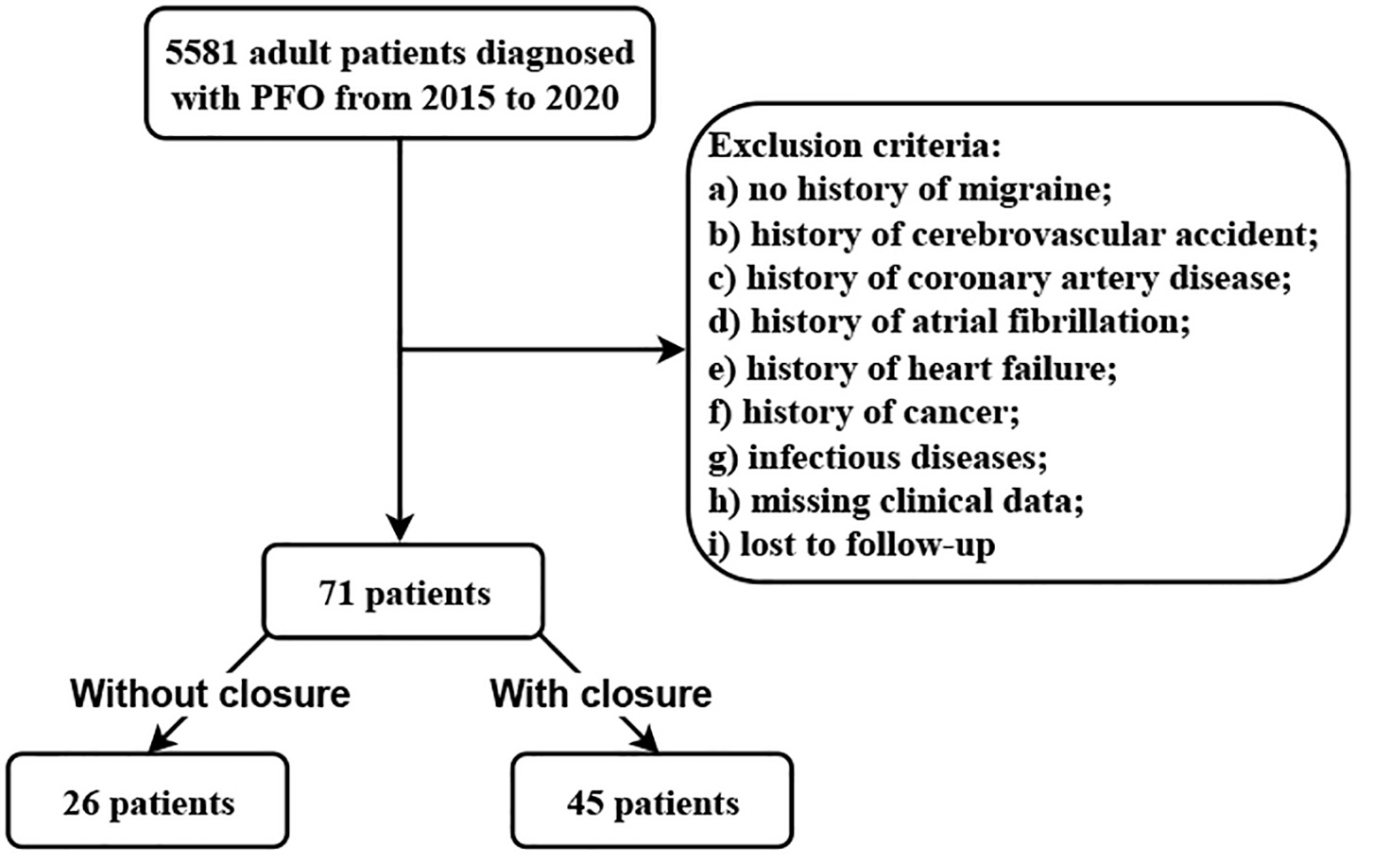
**Flow chart of the study population**. PFO, patent foramen ovale.

A total of 71 inpatients were included (Fig. 1). Of these, 45 underwent PFO closure, while 26 did not, either because they declined the procedure or the transseptal sheath could not traverse the atrial septum. The mean age was 43 years, and 57 patients were female. Baseline characteristics are shown in Table 1. Patients with PFO closure were younger, had a lower prevalence of diabetes, and demonstrated higher rates of migraine improvement.

**Table 1.** **Baseline characteristic**.

Variables	Without PFO closure (n = 26)	PFO closure (n = 45)	*p* value
Age (years)	48.0 ± 11.5	40.2 ± 12.6	0.011
Female (%)	24 (92.3%)	33 (73.3%)	0.053
Smoking (%)	1 (3.9%)	5 (11.1%)	0.537
Drinking (%)	1 (3.9%)	2 (4.4%)	1.000
Diabetes (%)	8 (30.8%)	4 (8.9%)	0.041
Hypertension (%)	7 (26.9%)	4 (8.9%)	0.092
Hyperlipidemia (%)	14 (53.8%)	15 (33.3%)	0.090
Hyperhomocysteinemia (%)	3 (11.5%)	4 (8.9%)	1.000
Migraine improvement (%)	14 (53.8%)	39 (86.7%)	0.002
BMI (kg/m^2^)	22.8 (19.9–25.7)	21.6 (19.7–24.9)	0.707
Admission SBP (mm Hg)	122 ± 12.1	122 ± 13.7	0.927
Admission DBP (mm Hg)	76.3 ± 9.4	76.8 ± 8.7	0.830
Admission HR (bpm)	79.6 ± 8.8	79.8 ± 9.2	0.925
LVEF (%)	67.3 (65.1–68.8)	66.0 (65.0–69.0)	0.788
ADLA (mm)	30.1 (28.0–33.5)	30.0 (27.0–32.0)	0.329
LVEDD (mm)	45.0 (44.0–47.5)	46.0 (43.0–47.0)	0.737
LDL-C (mmol/L)	2.85 (2.4–3.4)	2.70 (2.2–3.1)	0.550
TG (mmol/L)	1.36 (0.9–1.7)	1.35 (0.9–2.7)	0.853
TC (mmol/L)	4.16 (4.0–5.2)	4.16 (3.8–5.0)	0.363
HDL-C (mmol/L)	1.4 ± 0.3	1.3 ± 0.3	0.534
HCY (mmol/L)	10.1 (8.8–12.1)	10.4 (9.5–11.9)	0.872
FPG (mmol/L)	5.18 (4.9–5.7)	5.18 (4.7–5.4)	0.307
Creatinine (µmol/L)	58.8 (52.6–62.8)	58.2 (53.5–66.0)	0.891
WBC (×10^9^/L)	5.26 (5.0–6.2)	5.47 (4.8–6.9)	0.821
RBC (×10^9^/L)	4.4 ± 0.3	4.5 ± 0.3	0.518
PLT (×10^9^/L)	240 (212–260)	236 (210–272)	0.720
Hb (g/L)	135 (128–138)	136 (130–143)	0.296
ALT (U/L)	19.2 (13.2–24.8)	16.0 (11.0–19.2)	0.188
AST (U/L)	20.0 (18.2–24.2)	18.0 (15.0–21.0)	0.057

Mean (standard deviation) for variables with normal distribution. Medians (25%–75% percentiles) for variables with non-normal distribution. Abbreviations: BMI, body mass index; SBP, systolic blood pressure; DBP, diastolic blood pressure; HR, heart rate; LVEF, left ventricular ejection fraction; ADLA, anteroposterior diameter of left atrium; LVEDD, left ventricular end-diastolic dimension; LDL-C, low-density lipoprotein cholesterol; TG, triglyceride; TC, total cholesterol; HDL-C, high-density lipoprotein-cholesterol; HCY, homocysteine; FPG, fasting plasma glucose; WBC, white blood cell; RBC, red blood cell; PLT, platelet; Hb, hemoglobin; ALT, alanine transaminase; AST, aspartate transaminase.

In the PFO closure group, 20 patients (44.44%) experienced complete resolution of migraine. Among those without complete cure, 18 patients (40.00%) reported migraine improvement: 14 (31.11%) reported reductions in both frequency and severity, 2 (4.44%) reported a reduction in migraine score, and 2 (4.44%) reported improvement in frequency only. In patients without PFO closure, 2 (7.69%) patients had a remission of migraine, while 10 (38.46%) patients reported improvement: 5 (19.23%) reported reductions in both migraine frequency and severity score, 2 (7.69%) in migraine score only, and 3 (11.54%) in frequency only. As shown in Table 2, compared with patients without PFO closure, migraine frequency and severity score of patients with closure significantly decreased (*p* = 0.011, *p* < 0.001, respectively).

**Table 2.** **Comparison of the migraine before and after hospitalization**.

	Without PFO closure (n = 26)		PFO closure (n = 45)		Difference between PFO closure and PFO without closure (95% CI)	*p* value
	Before hospitalization	After hospitalization	Before hospitalization	After hospitalization		
Migraine frequency (events/month)	11.92 ± 1.89	3.46 ± 1.40	8.85 ± 1.43	6.62 ± 1.31	4.70 (1.13–8.28)	0.011
Subjective severity score	6.88 ± 0.37	2.54 ± 0.60	5.73 ± 0.31	4.77 ± 0.42	3.15 (1.92–4.38)	<0.001

Abbreviations: CI, confidence interval.

A few exceptions were noted. In the PFO closure group, one patient reported an increase in migraine frequency (from 2 to 8 per month), and another reported worsening severity (from 4 to 6). Importantly, one patient reported that 2 years after PFO closure, migraine frequency increased from 2 to 10 per month, and then after another 2 years, it decreased to 1 every 3 months. Another patient reported persistent but less frequent migraines for the first 2 years post-closure, followed by complete remission after another 2 years.

**Fig. 2.**
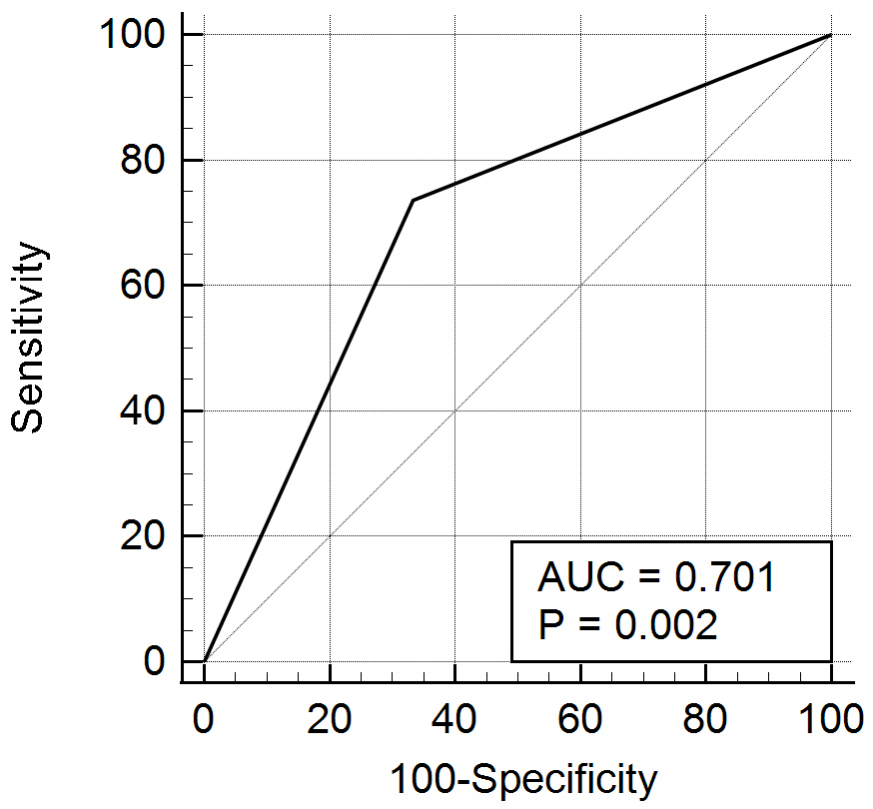
**ROC curve for predicting migraine improvement after PFO closure**. ROC, receiver operating characteristic; AUC, area under curve.

Univariate logistic regression analysis showed that PFO closure was significantly associated with migraine improvement (odds ratio (OR): 5.57, 95% confidence interval (CI): 1.76–17.68, *p* = 0.004). This association remained significant after adjustment for age and sex, and after further adjustment for significant covariates identified in Tables 1 and 3. ROC curve analysis confirmed the predictive ability of PFO closure for migraine improvement (*p* = 0.002; Fig. 2).

**Table 3.** **Odds ratios and 95% confidence intervals for migraine improvement**.

	PFO closure	
	OR (95% CI)	*p* value
Model 1	5.57 (1.76–17.68)	0.004
Model 2	6.08 (1.67–22.08)	0.006
Model 3	6.72 (1.75–25.78)	0.005

Model 1: Unadjusted. Model 2: Adjusted for age, gender. Model 3: Adjusted for age, gender, diabetes. Abbreviations: OR, odds ratio.

## 4. Discussion

Migraine is one of the most disabling neurological disorders worldwide, affecting approximately 6% of males and 18% of females, and is largely attributed to increased excitability of the central nervous system [33]. Several studies have reported an association between PFO and migraine; however, this relationship remains controversial, as the underlying mechanisms are not fully understood and clinical findings are inconsistent. While some studies suggest that percutaneous PFO closure alleviates migraines, others have reported no benefit.

In this study, we found that PFO closure significantly decreased both migraine frequency and severity. Logistic regression analyses revealed significant associations between PFO closure and migraine improvement. The relatively young age of our cohort reflects the exclusion of patients with a history of stroke, who are typically older. Previous research has shown that migraine frequency decreases with age, which may explain why younger patients were more likely to be hospitalized [34]. Our observed rates of complete remission (44.44%) and overall improvement (84.44%) are consistent with a previous meta-analysis, which reported that 46% of patients achieved complete cure and 83% experienced significant improvement after PFO closure [35].

Several observational studies and meta-analyses have demonstrated that PFO closure can relieve migraines [21, 22, 36, 37, 38]; however, previous comparative field studies failed to show an association between PFO and migraine. This inconsistency may be explained by poor echocardiographic assessment in earlier studies [7]. In our cohort, one patient reported that migraine relief was not apparent in the first year, and another patient reported that migraine was even more severe in the first 2 years. Previous randomized control trial (RCT) using Amplatzer occluders like in this study failed to show a statistically significant improvement in migraine after PFO closure when only their primary endpoints were considered. Yet, they showed significant improvements regarding most of their secondary endpoints [28, 29]. Importantly, those trials did not exclude patients with atrial fibrillation or a history of stroke, despite evidence that PFO prevalence is higher in patients with both stroke and migraine compared with those with migraine alone [16]. A previous retrospective analysis of 162 patients who underwent PFO closure to prevent recurrent stroke, including 57 with migraine, found that 56% had a complete cure and 14% had a significant reduction in migraine frequency [22]. The pooled analysis of the PRIMA and PREMIUM trials showed that PFO closure was safe and significantly reduced migraine frequency, resulting in a more complete cure [30]. Similarly, a meta-analysis of five RCTs and six observational studies demonstrated the efficacy of PFO closure in reducing monthly migraine attacks and days [39]. Taken together, our findings add meaningful evidence in determining the association between PFO closure and migraine, particularly because we excluded patients with a history of stroke.

Despite encouraging results, proving the efficacy of PFO closure in patients with migraine remains challenging. A key concern is why many observational studies have shown positive outcomes, whereas RCTs—especially double-blind designs—have produced negative or inconclusive results [40]. One explanation may be small sample sizes due to strict inclusion criteria, as many patients with migraine also have a history of stroke. Due to a poor sensitivity of the tests used for PFO screening (transthoracic echocardiography by inexperienced operators regarding PFO screening), PFOs were typically only found in about 15% people, meaning that about 10% of PFOs remained undetected in the control groups. This hid the deleterious effects of PFO in comparative field studies [41]. The prevalence of PFO is higher in patients with migraine and a history of stroke than in those with migraine alone [18]. Our study, a 5-year observational investigation of 71 patients, is among the few to exclude stroke cases, reducing recall bias that has limited earlier work. A previous observational study of 1101 stroke-free individuals found no significant association between PFO and migraine, with only 26 (2%) patients affected by both [26]. Similarly, a 1:1 matched case-control study of 288 stroke-free patients reported no significant difference in PFO prevalence between the migraine and control group [42]. Few studies, let alone RCTs, have excluded patients with a history of stroke. Taken together, our study is the first to demonstrate migraine improvement after PFO closure in stroke-free patients.

Notably, 2 patients in our study reported worsening migraines after closure, with 1 experiencing increased frequency and another increased severity. Previous studies have shown that up to 30% of patients develop new or transiently worsened migraines shortly after closure, though symptoms typically resolve within weeks [43, 44]. Theoretically, the therapeutic effect of PFO closure may be mechanistically immediate—by physically closing or substantially reducing the right-to-left shunt that facilitates paradoxical embolization and triggers migraines. Nickel hypersensitivity also represents a compelling and biologically plausible mechanism, as systemic nickel allergy has been associated with migraine pathogenesis and could potentially be exacerbated by a nickel-containing occluder device [45]. Nickel hypersensitivity exacerbates migraines through immune-driven neuroinflammation, direct neurostimulation by nickel ions, and amplified vascular dysregulation. Patients with nickel hypersensitivity are at significantly higher risk of developing device-related syndrome within 90 days of PFO closure, primarily driven by new/worsening migraines and palpitations [46].

Overall, PFO closure can significantly improve migraine outcomes. Interatrial communications beyond PFO, such as atrial septal defects, have also been associated with migraine, and their closure can also alleviate headaches [47]. In addition to its therapeutic effect on migraine, PFO closure provides lifelong mechanical prevention against paradoxical embolism, which may lead to potentially catastrophic outcomes, including stroke, myocardial infarction, peripheral or ocular ischemia, and mortality [48]. This protective benefit against embolic events is at least as clinically significant as its effect on migraines.

## 5. Limitations

This study had some limitations. First, migraine severity was assessed by self-reported frequency and intensity, which may introduce recall bias and lack standardization. Second, a previous study showed that the degree of right-to-left shunting of the PFO affects migraine aura frequency; however, in our study, PFO size and shunt severity were not evaluated. Third, the sample size was relatively small, limiting statistical power. Fourth, we did not stratify patients by migraine subtype (with vs. without aura), which may have influenced results. Fifth, as a single-center, retrospective study without a sham control group, our design cannot exclude placebo effects, a major limitation given the subjective nature of migraine outcomes. Sixth, data on migraine medications‒including type, dosage, and frequency‒were not systematically collected. Future multicenter, large-scale, prospective studies with detailed assessments are needed to validate our findings.

## 6. Conclusion

This retrospective study demonstrated a strong association between PFO and migraine. Percutaneous PFO closure significantly reduced migraine frequency and severity, with a substantial proportion of patients achieving complete remission. Although a minority experienced initial worsening, the long-term effects of PFO closure were generally beneficial.

## Availability of Data and Materials

The datasets used during the current study are available from the corresponding author on reasonable request.
